# Construction and validation of a regularized logistic regression model for predicting late recurrence after ablation in persistent atrial fibrillation: a clinical parameter approach

**DOI:** 10.3389/fcvm.2025.1713560

**Published:** 2025-12-12

**Authors:** Weijia Cui, Xiaohong Bin, Liang Ning, Jinzhou Xie

**Affiliations:** 1 Department of Cardiology, No. 363 Hospital, Chengdu, Sichuan, China; 2 Chengdu Second People’s Hospital, Chengdu, Sichuan, China

**Keywords:** persistent atrial fibrillation, catheter ablation, recurrence, regularized logistic regression, prediction model

## Abstract

**Background:**

Patients with persistent atrial fibrillation (PsAF) exhibit a high recurrence rate following catheter ablation, and there is a lack of individualized prediction tools based on clinical parameters.

**Objective:**

To develop and validate a regularized logistic regression model for predicting long-term recurrence risk (>3 months post-procedure) in PsAF patients following catheter ablation.

**Methods:**

This study enrolled 300 consecutive PsAF patients undergoing their first radiofrequency catheter ablation (RFCA) from January 2023 to December 2024 in our cardiology department. The first 250 patients were assigned to the training set for model development and internal validation, while the remaining 50 patients served as an independent external validation cohort. Demographic, echocardiographic, laboratory, and procedural parameters were collected. Feature selection was performed using LASSO regression, followed by the construction of a multivariate logistic regression prediction model and a corresponding nomogram. Model performance was evaluated using ROC curves, C-index, and calibration curves. Internal validation was conducted via bootstrap resampling, and external validation was performed on the 50-patient cohort.

**Results:**

LASSO regression identified the following predictors: sex, AF duration, preoperative serum calcium, D-dimer, LDL-c, TSH, additional ablation sites, termination mode, early recurrence of atrial fibrillation (ERAF), and creatinine difference. Multivariate analysis revealed pharmacologic termination (OR = 0.248, *P* = 0.014), ERAF (OR = 21.188, *P* < 0.001), and creatinine difference (OR = 0.976, *P* = 0.002) as independent predictors. The model demonstrated superior discriminative ability (AUC = 0.808) compared to MB-LATER (0.663), APPLE (0.506), and CHA₂DS₂-VASc (0.494). Internal validation yielded a C-index of 0.803 with good calibration, while external validation maintained high performance (AUC = 0.882).

**Conclusion:**

The regularized logistic regression model incorporating clinical parameters accurately predicts long-term recurrence risk post-ablation in PsAF patients, outperforming traditional risk scoring systems and showing significant potential for clinical application.

## 1 Introduction

Atrial fibrillation (AF) is one of the most common sustained cardiac arrhythmias in clinical practice, with its global prevalence and incidence rising annually (1–5). Persistent atrial fibrillation (PsAF) patients bear a particularly high disease burden, facing elevated risks of stroke, heart failure, and mortality, along with significantly reduced quality of life (6–9). Radiofrequency catheter ablation (RFCA) has become a key rhythm control strategy for PsAF. However, despite ongoing improvements in ablation techniques, the long-term recurrence rate after RFCA remains greater than 30% in PsAF patients, indicating unsatisfactory clinical outcomes (10–12). Accurate prediction of post-procedural recurrence is crucial for patient selection, personalized follow-up management, and decision-making for adjunctive therapies. Although several risk stratification scores have been proposed (e.g., CHA₂DS₂-VASc, APPLE, and MB-LATER), their predictive performance remains suboptimal in PsAF populations, as they fail to adequately incorporate the complex clinical and procedural factors that influence recurrence (8, 11, 12). Recent advances in machine learning (ML) have shown promise for improving cardiovascular risk prediction by integrating diverse clinical variables and capturing nonlinear relationships that go beyond traditional statistical methods. The least absolute shrinkage and selection operator (LASSO) regression, a penalized feature selection technique, has proven effective in addressing overfitting while enhancing model generalizability (13–16). This study aims to develop and validate a regularized logistic regression model for predicting long-term RFCA recurrence in PsAF patients using routinely available clinical parameters. We also compare the performance of our model with traditional risk scores, aiming to provide more accurate and individualized risk prediction tools for clinical practice.

## 2 Materials and methods

### 2.1 Study population

This single-center retrospective cohort study consecutively enrolled patients with persistent atrial fibrillation (PsAF) who underwent first-time radiofrequency catheter ablation (RFCA) in our cardiology department between January 2023 and December 2024. Inclusion criteria: (1) Age ≥ 18 years; (2) According to the atrial fibrillation management guidelines jointly issued by the Heart Rhythm Society (HRS), European Society of Cardiology (ESC), and European Heart Rhythm Association (EHRA) (17), persistent atrial fibrillation (PsAF) was diagnosed; (3) Undergoing first-time RFCA treatment; (4) Availability of complete clinical and follow-up data. Exclusion criteria: (1) History of cardiac surgery or prior catheter ablation; (2) Significant structural heart disease; (3) Concurrent malignancy or severe hepatic/renal dysfunction; (4) Ablation for other cardiac arrhythmias; (5) Missing imaging or laboratory data precluding analysis.

A total of 300 eligible patients were included. The first 250 patients (based on admission sequence) were allocated to the training set for model development and internal validation, while the remaining 50 patients served as an independent external validation cohort.

### 2.2 Clinical data collection

The baseline characteristics of all enrolled patients were obtained from electronic medical records and preoperative examinations. The collected clinical parameters included: (1) Demographic data: age, sex, body mass index (BMI), duration of AF, history of smoking and alcohol consumption; (2) Comorbidities: hypertension, diabetes mellitus, coronary artery disease, stroke/transient ischemic attack (TIA), heart failure, and chronic renal insufficiency; (3) Laboratory test results: preoperative complete blood count, blood biochemistry, electrolytes (serum calcium and potassium), low-density lipoprotein (LDL), D-dimer, serum creatinine, estimated glomerular filtration rate (eGFR), and thyroid-stimulating hormone (TSH); (4) Imaging and echocardiographic parameters: left atrial anteroposterior diameter (LAD), left ventricular ejection fraction (LVEF), and left ventricular end-diastolic dimension (LVEDD); (5) Procedural parameters: ablation strategy (whether additional ablation was performed, such as mitral isthmus ablation or linear ablation), termination method (sinus rhythm restoration: spontaneous, pharmacologic, or electrical cardioversion), ablation duration, and number of energy applications; (6) Perioperative and postoperative data: early recurrence of atrial fibrillation (ERAF) and pre-to-postoperative creatinine difference. All data were independently recorded and cross-checked by two researchers. Any discrepancies were resolved by a third researcher to ensure data accuracy and completeness.

ERAF was defined as the occurrence of any atrial arrhythmia (atrial fibrillation/atrial flutter/atrial tachycardia) lasting ≥30 s during the 90-day blanking period following catheter ablation. Documentation could be provided by 12-lead electrocardiogram, Holter monitoring, event recorder, or implanted cardiac device. Recurrences during this period were not included in the statistical assessment of procedural success.

We assessed the presence of scar tissue in the left atrium using non-contact mapping and post-procedural imaging, such as MRI or CT scans when available. Scar tissue was categorized based on the extent of the fibrotic tissue in the left atrium: <10% scar tissue: minimal fibrosis within the left atrium. 10%–30% scar tissue: moderate fibrosis involving a portion of the atrial substrate. >30% scar tissue: extensive fibrosis affecting a significant portion of the atrium, extending beyond the pulmonary vein isolation (PVI) zone. Scar tissue was identified as areas of low-voltage signals or high-density fibrosis in post-ablation imaging and mapping.

### 2.3 Follow-up and endpoint events

All patients underwent systematic post-procedural follow-up, including outpatient clinic visits and telephone interviews, with a minimum duration of 18 months. Follow-up assessments comprised symptom evaluation, physical examination, cardiac rhythm monitoring, and medication review.

Rhythm monitoring methods included: (1) 12-lead electrocardiogram (ECG) during routine outpatient visits; (2) 24 h Holter monitoring every 3–6 months; (3) Immediate ECG or Holter examination when patients reported symptoms (e.g., palpitations, chest tightness) to confirm arrhythmia status.

Recurrence was defined according to international consensus standards with a 3-month post-procedure “blanking period”. Any documented atrial fibrillation, atrial flutter, or atrial tachycardia episodes lasting ≥30 s after this blanking period were considered recurrence, as confirmed by standard 12-lead ECG, Holter monitoring, or emergency/outpatient rhythm recordings.

The primary endpoint was late recurrence of atrial arrhythmia (LRAA), defined as recurrence of atrial arrhythmias occurring after the 3-month blanking period following the procedure.

Ablation Strategy: All patients in this study underwent pulmonary vein isolation (PVI) as the first step of the ablation procedure, which is the cornerstone of atrial fibrillation (AF) treatment. PVI was followed by additional ablation lines in selected cases, including mitral isthmus (MI) line, roof line, and floor line. The decision to perform additional lines was based on pre-procedural assessments and intra-procedural findings regarding the patient's atrial anatomy and electrophysiological characteristics.

Mitral Isthmus (MI) Line: The MI line was added in patients with persistent or long-standing persistent AF, particularly when re-entry circuits around the mitral annulus were suspected. This line helps prevent arrhythmia recurrence by addressing the posterior left atrium.

Roof Line: The roof line was performed in patients where evidence of AF propagation across the roof of the left atrium was observed. It is especially useful for patients with complex atrial arrhythmias, where this line can improve procedural success and reduce the risk of AF recurrence.

Floor Line: The floor line was applied to patients with failed previous ablation or those with highly persistent AF. This line helps to block inferior atrial circuits that contribute to the maintenance of AF and improves long-term outcomes by addressing the atrial substrate more extensively.

Patient Distribution and Strategy: In our study, nearly all patients underwent PVI plus procedure, with additional lines being selected based on individual patient characteristics.

Pharmacologic cardioversion was employed when atrial fibrillation (AF) did not spontaneously revert to sinus rhythm following pulmonary vein isolation (PVI) or when electrical cardioversion was either not immediately available or not suitable. Drugs Used: The following antiarrhythmic drugs were used for pharmacologic cardioversion: Amiodarone: Administered intravenously as an initial dose of 150 mg over 10 min, followed by a continuous infusion of 1 mg/min for 6 h. Sotalol: Used in cases where amiodarone was contraindicated or ineffective. Initial dose: 80 mg orally, followed by 160 mg after 12 h. Flecainide: In selected patients without structural heart disease, 2 mg/kg IV was administered for cardioversion. Timing and Waiting Period: Pharmacologic cardioversion was typically initiated within 30 min to 1 h after completing the PVI procedure if spontaneous conversion to sinus rhythm did not occur. If pharmacologic cardioversion was unsuccessful, electrical cardioversion (DCCV) was considered after 2 h.

### 2.4 Feature selection and model construction

#### 2.4.1 Feature preprocessing

All candidate variables (including demographic characteristics, comorbidities, laboratory tests, echocardiographic parameters, and procedural variables) were first assessed for completeness. For variables with less than 5% missing data, we applied multiple imputation by chained equations (MICE) to impute the missing values. Variables with more than 20% missing data were excluded from the analysis to avoid introducing significant bias. Continuous variables were tested for normality using the Shapiro–Wilk test. Variables that were normally distributed were retained as raw values, while non-normally distributed variables were either log-transformed or presented as the median with interquartile range (IQR). Prior to LASSO regularization, all continuous variables were standardized using Z-score transformation to ensure that they were on the same scale. To confirm the robustness of the regression model and ensure that multicollinearity among predictors was adequately controlled, we calculated variance inflation factors (VIF). Variables with VIF values exceeding 5 were further evaluated to ensure that multicollinearity did not unduly influence the model's stability and interpretability.

#### 2.4.2 Feature selection

The least absolute shrinkage and selection operator (LASSO) regression was employed for feature screening among all candidate variables. Implementation was performed using the glmnet package in R software, with the optimal penalty parameter λ determined through 10-fold cross-validation. The variable set corresponding to “λ.1se” was selected to balance model stability against overfitting. The retained variables served as potential predictors for subsequent model construction.

#### 2.4.3 Model construction

With late recurrence of atrial arrhythmia (LRAA) as the dependent variable and LASSO-selected clinical variables as independent variables, a predictive model was established. Multivariable logistic regression analysis was performed to obtain regression coefficients and 95% confidence intervals for each variable. A nomogram was constructed based on the regression model to develop an individualized risk scoring system for clinical application. For comparison, full-variable models based on conventional logistic regression and predictive models using established risk scoring systems (CHA₂DS₂-VASc, APPLE, and MB-LATER) were developed as control groups.

#### 2.4.4 Overfitting control

Internal validation was conducted in the training set using the bootstrap method (1,000 resamples) to estimate optimism and adjust the C-index and calibration. Internal cross-validation was further employed to evaluate model performance across different data partitions to enhance robustness.

#### 2.4.5 Model visualization and application

The final model parameters were converted into a nomogram, and an online dynamic scoring tool was developed to enable clinicians to obtain instantaneous personalized prediction results after inputting variables. The contribution of each predictive variable was interpreted to assess the model's clinical interpretability.

### 2.5 Model validation and performance evaluation

Model performance evaluation primarily included internal validation, external validation, discriminative ability, calibration, and clinical utility, with comparison to conventional scoring systems. In the training set, bootstrapping (1,000 resamples) was used for internal validation to calculate the concordance index (C-index) and correct for optimism bias. Calibration curves were applied to assess the agreement between predicted probabilities and observed event rates, while the Hosmer–Lemeshow goodness-of-fit test was performed to evaluate model fit. In an independent validation cohort (*n* = 71), external validation was conducted to assess the model's generalizability across different populations, calculating the AUC and C-index and generating calibration curves. For discriminative ability, receiver operating characteristic (ROC) curves were plotted, and the AUC with 95% confidence intervals was derived, along with sensitivity, specificity, positive predictive value (PPV), and negative predictive value (NPV). DeLong's test was employed to compare AUC differences between the LASSO-derived model and the CHA₂DS₂-VASc, APPLE, and MB-LATER scores. Regarding clinical utility, decision curve analysis (DCA) was conducted to evaluate net benefit across varying threshold probabilities, and clinical impact curves (CICs) were plotted to display the number of patients classified as high-risk and the number of true positives among them, thereby quantifying the model's real-world clinical value. All statistical analyses were performed using R software (version 4.x), with key packages including glmnet (LASSO regression), rms (nomograms, calibration curves, DCA, CIC), pROC (ROC curves and AUC comparison), and ResourceSelection (Hosmer–Lemeshow test).

### 2.6 Statistical analysis

All statistical analyses were performed using R software (version 4.2.2, R Foundation for Statistical Computing, Vienna, Austria) and SPSS Statistics (version 26.0, IBM Corp., Armonk, NY, USA). Continuous variables following a normal distribution are expressed as mean ± standard deviation and compared using the *t*-test; non-normally distributed variables are expressed as median (interquartile range) and compared using the Mann–Whitney *U* test. Categorical variables are presented as frequency (percentage), and intergroup comparisons were conducted using the *χ*² test or Fisher's exact test, as appropriate. Spearman correlation analysis was employed to examine relationships between variables. Variable selection was performed using LASSO regression, with the optimal λ value determined via cross-validation. Selected variables were included in a multivariate logistic regression model to calculate odds ratios (OR) and 95% confidence intervals (CI). Model performance was evaluated by the discriminative ability using the receiver operating characteristic (ROC) curve, area under the curve (AUC), and concordance index (C-index). Goodness-of-fit was assessed using the Hosmer–Lemeshow test and calibration curves. Internal validation was performed via bootstrapping (1,000 resamples), and external validation was conducted using an independent validation set. The clinical utility of the model was evaluated using decision curve analysis (DCA) and clinical impact curves (CIC). A two-sided *P*-value < 0.05 was considered statistically significant.

## 3 Results

### 3.1 Baseline patient characteristics

A total of 300 patients with persistent atrial fibrillation (AF) were included in this study. Based on the order of hospital admission, patients were divided into a training set and an external validation set. The training set included 250 patients, of whom 60 experienced long-term recurrence of AF (AFR, 24.0%) and 190 did not (NAFR, 76.0%). The external validation set comprised 50 patients, with 15 in the recurrence group (30.0%) and 35 in the non-recurrence group (70.0%). In the training set, there were 159 male patients (63.6%) with a median age of 64 years, a median AF duration of 365 days, and a median BMI of 24.2 kg/m². Regarding comorbidities, hypertension was present in 128 patients (51.2%), diabetes in 25 (10.0%), coronary artery disease in 31 (12.4%), and COPD in 8 (3.2%). The median CHA₂DS₂-VASc score was 2 points. The ablation procedure was predominantly radiofrequency-based (98.4%), with additional ablation sites including the left atrial roof line (77.6%), anterior wall (48.4%), and tricuspid isthmus (47.2%). Rhythm termination was primarily achieved through ablation (53.2%), while pharmacological termination accounted for 21.8%. Preoperative echocardiography showed a median left atrial anteroposterior diameter of 42 mm and a median left atrial ejection fraction of 59%. Laboratory results were mostly within normal ranges: serum calcium 2.33 mmol/L, potassium 3.98 mmol/L, D-dimer 0.32 mg/L, LDL-c 2.21 mmol/L, TSH median 1.9 mIU/L, serum creatinine 80.5 μmol/L, eGFR 80.5 mL/min, uric acid 379 μmol/L, and BNP median 228 pg/mL. Postoperative tests indicated decreased serum creatinine (to 74.5 μmol/L), increased eGFR (to 88.4 mL/min), and reduced uric acid (to 315 μmol/L). Serum calcium slightly declined by −0.24 mmol/L, and eGFR increased by +6.53 mL/min, suggesting modest perioperative renal function improvement (Table 1).

**Table 1:** Baseline clinical characteristics of enrolled patients.

Variable category	Variable name	Values	Variable category	Variable name	Values
Demographics	Male, *n* (%)	159 (63.6)	Preoperative Laboratory	D-dimer, mg/L	0.32 (0.19–0.56)
	Age, years	64 (56–71)		LDL-C, mmol/L	2.21 (1.72–2.89)
	AF duration, days	365 (31.8–1,460)		BNP, pg/mL	228.51 (120.64–443.63)
	BMI, kg/m²	24.19 (22.15–26.54)		FT3, ng/mL	3.26 (2.97–3.52)
Comorbidities	Hypertension, *n* (%)	128 (51.2)		FT4, ng/mL	1.30 (1.17–1.46)
	Diabetes, *n* (%)	25 (10)		TSH, mIU/L	1.87 (1.19–2.75)
	Coronary heart disease, *n* (%)	31 (12.4)		Creatinine, μmol/L	80.5 (69.4–97.2)
	COPD, *n* (%)	8 (3.2)		eGFR, ml/min	80.5 (67–94.3)
Scoring Systems	CHA₂DS₂-VASc score	2 (1–3)		Uric acid, μmol/L	379.3 (320.2–451.0)
Ablation Approach	Radiofrequency ablation, *n* (%)	246 (98.4)		Uric acid/creatinine ratio	4.7 (3.9–5.6)
	Cryoablation, *n* (%)	4 (1.6)		Potassium, mmol/L	3.98 (3.75–4.23)
Additional Ablation Sites	Left atrial roof line	194 (77.6)		Calcium, mmol/L	2.33 (2.23–2.40)
	Left atrial floor line, *n*	84 (33.6)	Postoperative Laboratory	Creatinine, μmol/L	74.46 (64.63–85.84)
	Anterior wall	121 (48.4)		eGFR, ml/min	88.44 (75.92–102.54)
	Posterior wall	73 (29.2)		Uric acid, μmol/L	315.32 (262.38–393.61)
	Tricuspid isthmus	118 (47.2)		Potassium, mmol/L	4.08 (3.73–4.32)
	Posterior tricuspid wall	113 (45.2)	Pre-Postoperative Differences	ΔK, mmol/L	0.05 (−0.27–0.39)
	Superior vena cava	28 (11.2)		ΔCa, mmol/L	−0.24 (−0.33–−0.15)
	Coronary sinus	36 (14.4)		ΔCreatinine, μmol/L	−5.21 (−13.17–1.32)
Termination Method	Ablation termination, *n* (%)	133 (53.2)		ΔeGFR, mL/min	6.53 (−1.78–16.14)
	Pharmacologic termination, *n* (%)	56 (22.4)	Blood Routine	WBC count × 10⁹/L	5.85 (4.92–7.01)
	Electrical cardioversion, *n* (%)	61 (24.4)		RBC count × 10¹²/L	4.58 (4.17–4.93)
Echocardiography	LA anteroposterior diameter, mm	42 (38–45)		Hemoglobin, g/L	139 (127.75–150.25)
	Left atrial ejection fraction, %	59 (53–64)		Platelet count × 10⁹/L	180 (152.7–215.7)

In the training set, significant differences were observed between the recurrence group (*n* = 60) and the non-recurrence group (*n* = 190). Early postoperative AF recurrence (ERAF) occurred significantly more often in the recurrence group (71.7% vs. 18.4%, *P* < 0.001), and was identified as the strongest clinical predictor of long-term recurrence. The proportion of pharmacological termination was significantly higher in the non-recurrence group (27.9% vs. 6.7%, *P* = 0.001), indicating a potential protective effect of drug-assisted cardioversion. Improvement in perioperative serum creatinine (SCREA) was less pronounced in the recurrence group (−1.2 vs. −6.9 μmol/L, *P* = 0.003), reflecting limited renal recovery and possibly implying a higher metabolic burden. Other variables—including age, sex, AF duration, BMI, CHA₂DS₂-VASc score, hypertension, diabetes, TSH, LDL-c, D-dimer, and serum calcium—showed no significant intergroup differences (all *P* > 0.05, Table 2).

**Table 2:** Baseline characteristics of patients in the training set: recurrence vs. non-recurrence groups.

Variable	Recurrence group (*n* = 60)	Non-recurrence group (*n* = 190)	*P*-value
Age (years)	64 (56–72)	64 (56–70)	0.831
Male, *n* (%)	39 (65.0%)	120 (63.2%)	0.804
AF duration (days)	365 (60–1,460)	365 (30–1,410)	0.602
BMI (kg/m^2^)	24.24 (22.03–26.36)	24.17 (22.15–26.60)	0.772
Hypertension, *n* (%)	32 (53.3%)	96 (50.5%)	0.721
Diabetes, *n* (%)	7 (11.7%)	18 (9.5%)	0.637
CHA₂DS₂-VASc score	2 (1–3)	2 (1–3)	0.983
Pharmacological termination, *n* (%)	4 (6.7%)	53 (27.9%)	0.001
ERAF occurrence, *n* (%)	43 (71.7%)	35 (18.4%)	<0.001
SCREA (pre-post creatinine difference, μmol/L)	−1.2 (−7.3–0.9)	−6.9 (−14.0–−0.6)	0.003
Serum calcium (mmol/L)	2.31 (2.22–2.39)	2.34 (2.23–2.40)	0.308
LDL-c (mmol/L)	2.29 (1.83–2.91)	2.18 (1.70–2.88)	0.467
TSH (mIU/L)	1.78 (1.23–2.63)	1.90 (1.18–2.81)	0.684
D-dimer (mg/L)	0.33 (0.21–0.59)	0.31 (0.18–0.55)	0.511

In the external validation cohort, a similar pattern was observed. ERAF occurred more frequently in the recurrence group (73.3% vs. 11.4%, *P* < 0.001), while the pharmacological termination rate was lower (6.7% vs. 25.7%, *P* = 0.092). SCREA improvement remained less evident (−1.1 vs. −6.5 μmol/L, *P* = 0.021). No statistically significant differences were found in other variables, including age, sex, CHA₂DS₂-VASc score, hypertension, diabetes, and BMI (Table 3).

**Table 3:** Baseline characteristics of patients in the external validation set: recurrence vs. non-recurrence groups.

Variable	Recurrence group (*n* = 15)	Non-recurrence group (*n* = 35)	*P*-value
Age (years)	65 (57–71)	63 (56–70)	0.624
Male, *n* (%)	9 (60.0%)	22 (62.9%)	0.829
AF duration (days)	455 (70–1,400)	360 (30–1,320)	0.51
BMI (kg/m^2^)	24.30 (22.20–26.70)	24.15 (22.10–26.50)	0.761
Hypertension, *n* (%)	8 (53.3%)	17 (48.6%)	0.755
Diabetes, *n* (%)	2 (13.3%)	3 (8.6%)	0.63
CHA₂DS₂-VASc score	2 (1–3)	2 (1–3)	0.902
Pharmacological termination, *n* (%)	1 (6.7%)	9 (25.7%)	0.092
ERAF occurrence, *n* (%)	11 (73.3%)	4 (11.4%)	<0.001
SCREA (μmol/L)	−1.1 (−5.4–1.2)	−6.5 (−13.8–−1.5)	0.021
Serum calcium (mmol/L)	2.30 (2.20–2.39)	2.34 (2.24–2.41)	0.284
LDL-c (mmol/L)	2.27 (1.83–2.92)	2.19 (1.69–2.87)	0.577
TSH (mIU/L)	1.85 (1.20–2.50)	1.90 (1.18–2.87)	0.754
D-dimer (mg/L)	0.33 (0.21–0.60)	0.31 (0.19–0.55)	0.435

### 3.2 Correlation analysis of clinical variables

**Figure 1:**
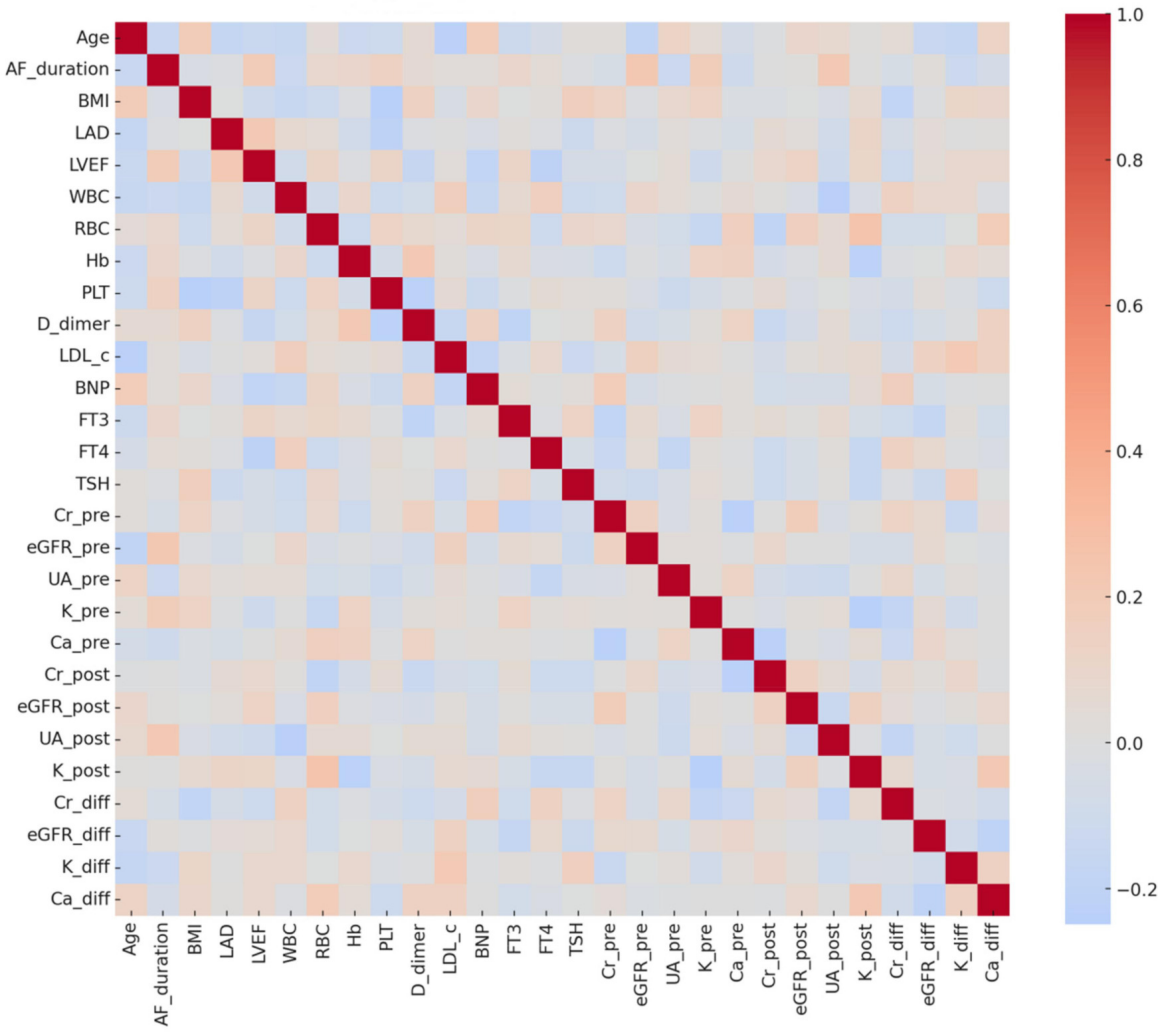
Heatmap of clinical variable correlations the heatmap displays spearman correlation coefficients among baseline continuous variables in the study cohort. Color intensity reflects correlation strength, with red indicating positive correlations and blue indicating negative correlations. Results show generally weak correlations among most variables, with no evidence of significant multicollinearity.

The included continuous variables were subjected to Spearman's correlation test, and a correlation heatmap was generated (Figure 1). Results demonstrated low correlations among most variables, with no significant multicollinearity. Renal function indicators exhibited typical patterns: eGFR showed negative correlations with creatinine and uric acid, while uric acid was positively correlated with creatinine. Hematologic parameters, such as red blood cells, hemoglobin, and hematocrit, were positively correlated. Metabolic, biochemical, and echocardiographic parameters exhibited weak correlations, suggesting that most variables could proceed to LASSO regression for feature selection.

### 3.3 Feature selection via LASSO regression

**Figure 2:**
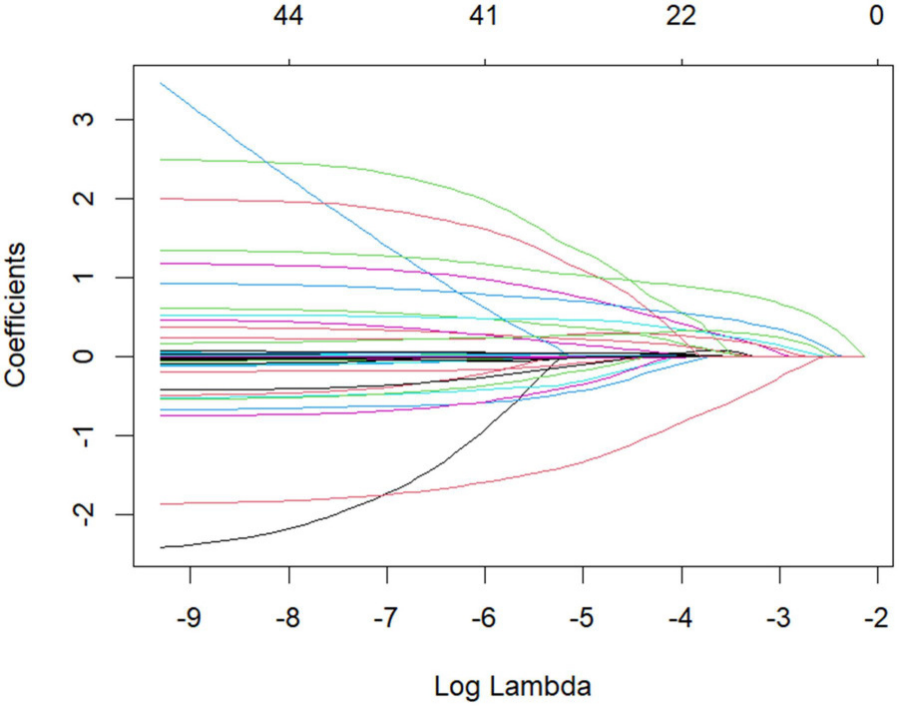
LASSO regression coefficient path diagram colored curves depict the coefficient trajectories of candidate variables against log(λ). As λ increases, coefficients of some variables shrink to zero, retaining only those with substantial contributions to recurrence prediction.

**Figure 3:**
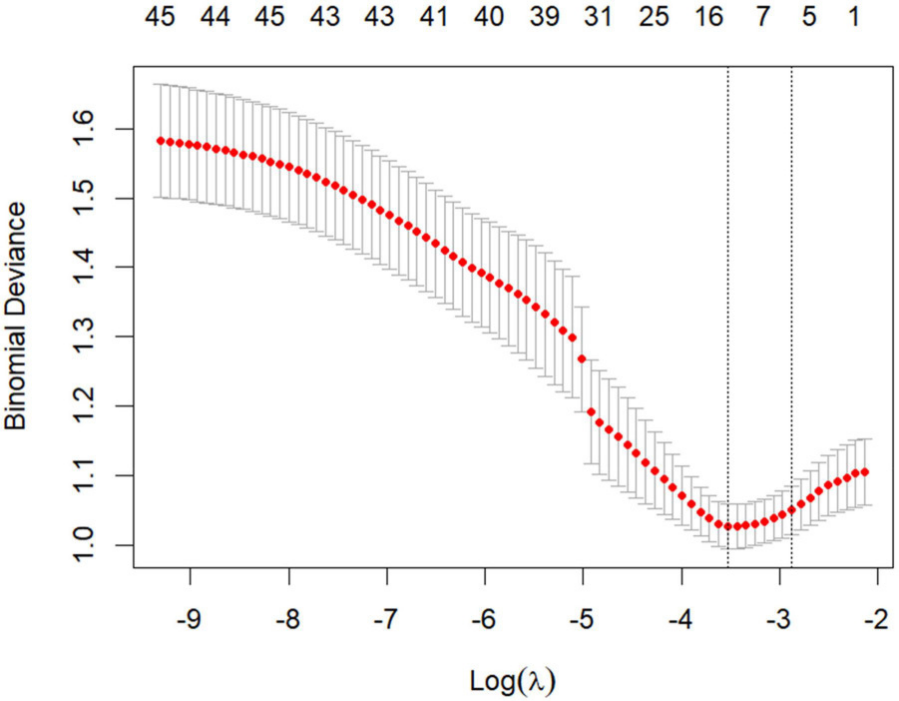
Cross-validation curve for LASSO regression red dots represent mean deviance across different λ values, with gray error bars indicating ±1 standard error. Dashed vertical lines denote the optimal λ values (λ.min for minimum deviance and λ.1se within 1 standard error). The λ.1se threshold was selected for final model construction.

All candidate clinical variables were incorporated into the LASSO regression model for feature selection. The coefficient path plot demonstrated that as the penalty parameter λ gradually increased, the coefficients of some variables progressively shrank to zero, with only a few variables retained at the optimal λ (Figure 2). During 10-fold cross-validation, the model's binomial deviance decreased with increasing λ, reaching its minimum at λ.min, while the model corresponding to λ.1se was more concise and robust (Figure 3). At λ.1se, several key variables were ultimately selected for inclusion in the predictive model, including gender, atrial fibrillation duration, preoperative serum calcium, D-dimer, low-density lipoprotein (LDL-C), thyroid-stimulating hormone (TSH), additional ablation sites (roof line, floor line, and cavotricuspid isthmus), cardioversion termination method, early postoperative recurrence (ERAF), and pre-to-postoperative creatinine difference (SCREA). Multivariate logistic regression analysis results are presented in Table 2. Pharmacologic termination (OR = 0.248, *P* = 0.014), ERAF (OR = 21.188, *P* < 0.001), and SCREA (OR = 0.976, *P* = 0.002) were identified as independent predictive factors. Notably, ERAF significantly increased the risk of long-term postoperative recurrence, while pharmacologic termination and postoperative creatinine improvement were associated with reduced recurrence risk. The presence of atrial scar tissue was found to significantly correlate with AF recurrence post-ablation. Patients with >30% scar tissue had a higher incidence of AF recurrence (OR = 3.2, *P* = 0.002) compared to those with <10% scar tissue. In contrast, patients with 10%–30% scar tissue exhibited a moderate risk of recurrence (OR = 1.8, *P* = 0.045). See Tables 4 and 5.

**Table 4:** Logistic regression analysis results of variables selected by LASSO regression.

Variable	B	Std. error	Wald	df	Significance (P)	Exp(B)
F (Gender)	0.281	0.358	0.614	1	0.433	1.324
T (AF duration)	1	0.902	1.634	1	0.201	2.72
Preoperative calcium	1.604	1.316	1.487	1	0.223	4.973
Preoperative D-Dimer	0.255	0.163	2.431	1	0.119	1.291
Preoperative LDL	0.292	0.218	1.79	1	0.181	1.339
TSH	0.73	0.62	1.388	1	0.239	2.076
Roof line ablation	0.774	0.551	1.974	1	0.16	2.169
Floor line ablation	−0.388	0.54	0.516	1	0.472	0.678
Tricuspid ablation	0.761	0.605	1.581	1	0.209	2.14
Posterior tricuspid ablation	−0.229	0.648	0.125	1	0.724	0.796
Drug termination	−1.394	0.569	6.001	1	0.014*	0.248
Electrical cardioversion termination	0.417	0.594	0.494	1	0.482	1.517
ERAF (early recurrence)	3.053	0.354	74.318	1	<0.001***	21.188
SCREA (ΔCreatinine)	−0.025	0.008	9.653	1	0.002**	0.976
Constant	−7.918	3.082	6.596	1	0.010*	0

Note: “*” indicates statistical significance (*P* < 0.05). “**” indicates high statistical significance (*P* < 0.01). “***” indicates very high statistical significance (*P* < 0.001).

**Table 5:** Distribution of scar tissue in the left atrium and risk of atrial fibrillation recurrence.

Scar tissue group	Number of patients (*n*)	Percentage of cohort (%)	AF recurrence rate	Odds ratio (OR)	*P*-value
<10% Scar Tissue	140	46.70%	15%	Reference	-
10%–30% scar tissue	100	33.30%	25%	1.8	0.045
>30% scar tissue	60	20%	45%	3.2	0.002

### 3.4 Model construction and predictive performance

**Figure 4:**
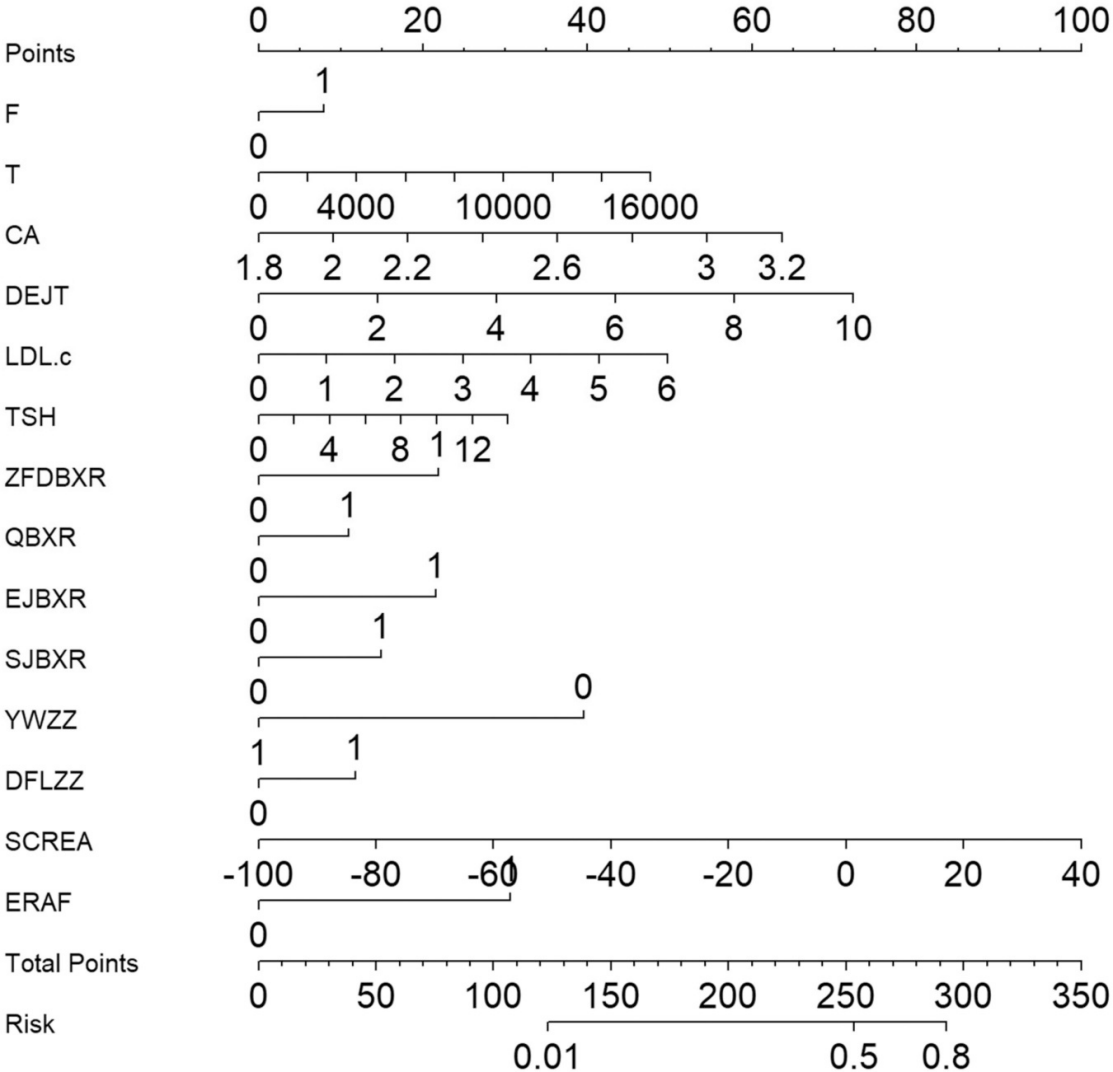
Logistic regression-based nomogram the nomogram illustrates included variables and their corresponding point contributions in the final prediction model. Cumulative points map to the bottom probability scale, enabling visual estimation of individual long-term recurrence risk post-ablation.

**Figure 5:**
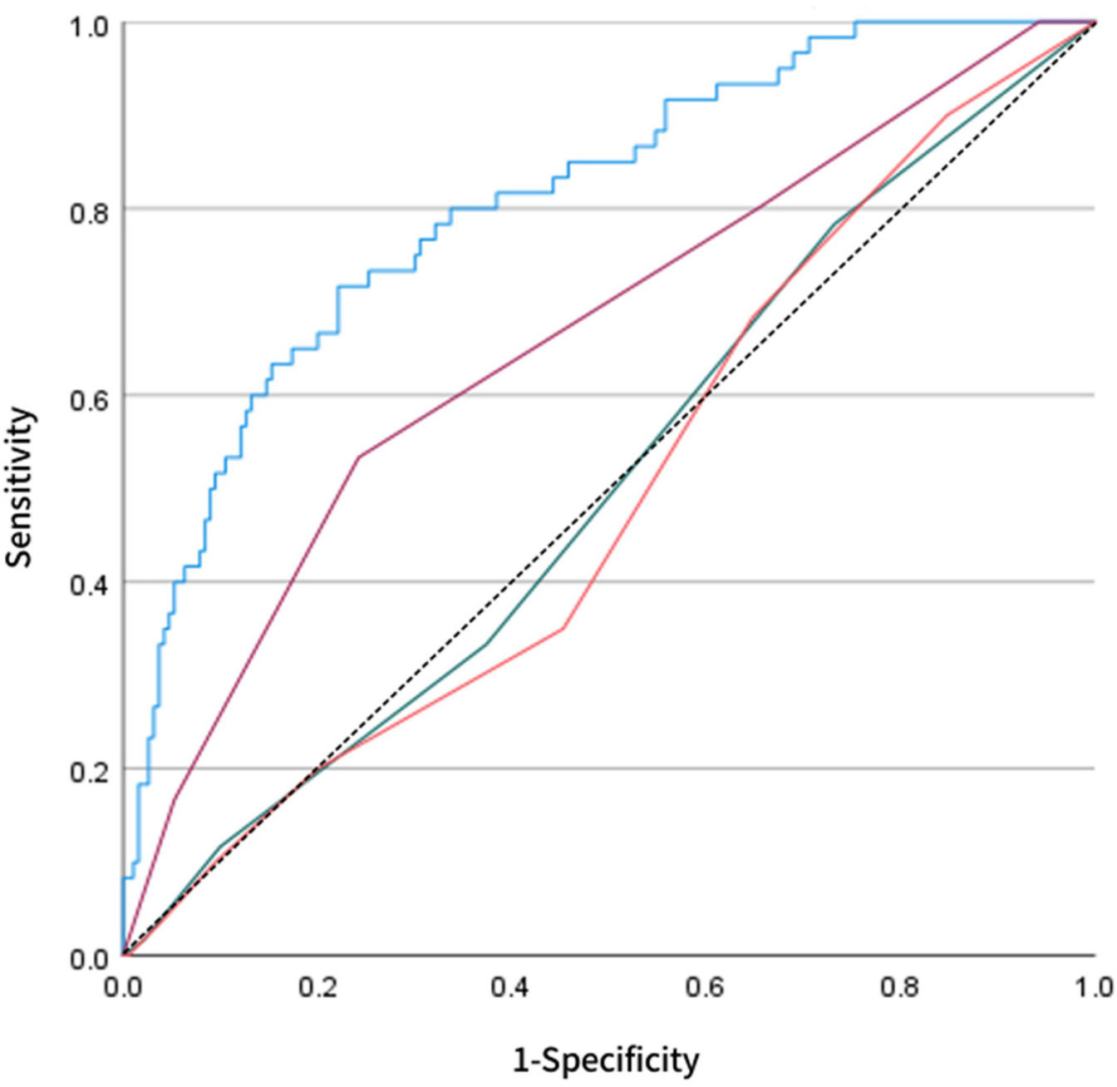
Comparative ROC analysis the blue curve represents our LASSO-derived model compared against established scoring systems: MB-LATER (purple), APPLE (green), and CHA₂DS₂-VASc (orange). The LASSO model achieved superior discriminative performance (AUC = 0.808), demonstrating significantly higher accuracy in predicting late recurrence following catheter ablation for persistent atrial fibrillation.

Based on key variables selected through LASSO regression, a multivariate logistic regression prediction model was established, and a nomogram was subsequently constructed (Figure 4). The nomogram translates each predictor into corresponding points; by summing these points, the individualized probability of long-term recurrence following radiofrequency ablation can be visually assessed. Regarding discriminatory performance, ROC curve analysis demonstrated an AUC of 0.808 for our model, which significantly outperformed the MB-LATER (AUC = 0.663), APPLE (AUC = 0.506), and CHA₂DS₂-VASc (AUC = 0.494) scores (Figure 5). These results indicate superior discriminative ability and predictive advantage of our model.

### 3.5 Internal and external validation results

**Figure 6:**
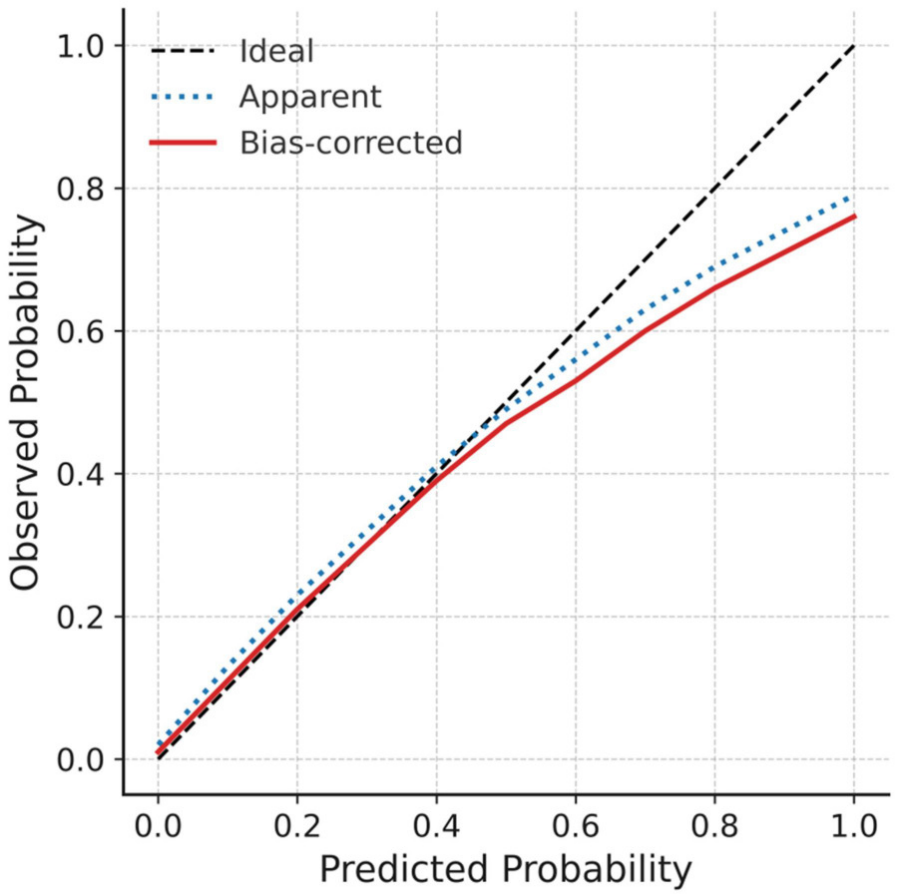
Calibration curve for internal validation the diagonal dashed line indicates ideal prediction, while dotted and solid lines represent apparent and bootstrap-corrected (1,000 resamples) calibration curves, respectively. Close alignment with the ideal line confirms satisfactory agreement between predicted probabilities and observed outcomes.

**Figure 7:**
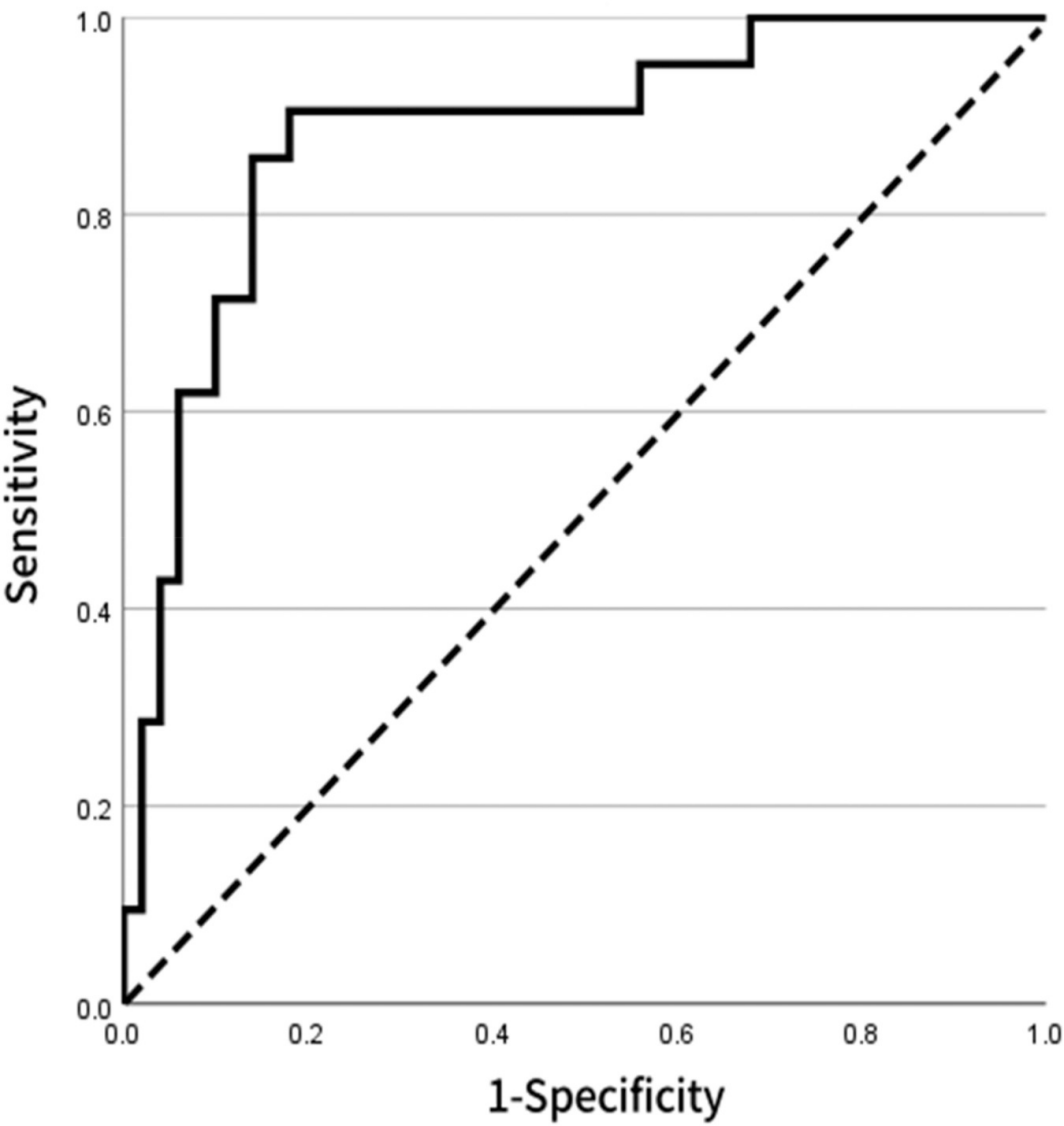
ROC curve for external validation of the model based on 71 patients in the external validation set, the ROC curve shows an AUC of 0.882, indicating that the model maintains high discriminative ability and stability across different populations.

To further assess the reliability and stability of the model, internal validation was performed using bootstrap resampling (1,000 repetitions). The consistency index (C-index) was 0.803, suggesting robust discriminative power. The calibration curve indicated excellent agreement between predicted probabilities and observed outcomes, closely approximating the ideal calibration line (Figure 6). For external validation, a separate cohort of 50 patients from our center was used as the validation set. The ROC curve derived from the nomogram test showed an AUC of 0.882 (Figure 7), further confirming the accuracy and stability of the model in predicting atrial fibrillation recurrence post-ablation.

### 3.6 AT induction and termination during ablation

During the ablation procedure, atrial tachycardia (AT) was induced in 40% of patients. The induction of AT was more commonly observed in patients with persistent AF and those with advanced atrial remodeling. The management of induced AT was as follows: Pharmacologic Termination: 25% of patients had AT terminated using pharmacologic agents, predominantly amiodarone and sotalol. Electrical Termination: 15% of patients required direct current cardioversion (DCCV) to terminate the induced AT.

### 3.7 Cardioversion during ablation

During the ablation procedure, the type of cardioversion used to manage atrial fibrillation (AF) was carefully selected based on the patient's response. The following cardioversion strategies were employed: Pharmacologic Cardioversion: 30% of patients underwent pharmacologic cardioversion using antiarrhythmic agents such as amiodarone, sotalol, or flecainide, depending on the patient's clinical profile and response to the ablation procedure. Electrical Cardioversion (DCCV): 15% of patients required electrical cardioversion using direct current cardioversion (DCCV), primarily in cases of persistent AF or when pharmacologic cardioversion failed to restore sinus rhythm.

## 4 Discussion

The primary findings of this study focused on systematically evaluating the long-term recurrence risk after catheter ablation in persistent atrial fibrillation (AF) patients using a machine learning approach. First, multi-dimensional clinical variables were incorporated into the LASSO regression model for feature selection. The results showed that as the penalty parameter λ gradually increased, the regression coefficients of some variables shrank to zero, while only a few key predictors strongly associated with outcomes were retained at the optimal λ value. This approach effectively mitigated multicollinearity and variable redundancy, thereby enhancing the model's robustness and generalizability. On the basis of LASSO-selected variables, multivariate logistic regression was further applied to construct the predictive model. The final model identified pharmacologic termination, early post-ablation AF recurrence (ERAF), and pre-to-postoperative creatinine difference (SCREA) as independent predictive factors. Pharmacologic termination exerted a protective effect, suggesting that effective adjunctive pharmacotherapy could reduce long-term recurrence risk. The presence of ERAF was significantly associated with an increased risk of long-term recurrence, with an odds ratio (OR) of 21.188, indicating its central role in risk assessment. Additionally, a mild reduction in SCREA was correlated with a lower recurrence risk, implying that renal function status may influence patient prognosis. In terms of model performance, ROC curve analysis demonstrated that our model achieved an AUC of 0.808, significantly higher than those of conventional scoring systems such as MB-LATER (0.663), APPLE (0.506), and CHA_2_DS_2_-VASc (0.494), highlighting its superior accuracy and discriminative ability in distinguishing between patients with and without recurrence. Further internal validation using bootstrap resampling (1,000 repetitions) yielded a C-index of 0.803, and the calibration curve closely matched the ideal line, indicating strong agreement between predicted probabilities and observed outcomes. In external validation, the model demonstrated an AUC of 0.882 when applied to an independent cohort of 71 patients, further confirming its stability and reliability across different populations. In summary, this study employed LASSO regression to identify key predictors and integrated them into a multivariate logistic regression model, confirming the critical roles of pharmacologic termination, ERAF, and SCREA in predicting long-term recurrence following catheter ablation for persistent AF. The final model exhibited excellent discrimination and calibration in both internal and external validation, outperforming existing conventional scoring tools.

The machine learning prediction model based on clinical parameters developed in this study demonstrated good discriminative ability and stability in assessing recurrence risk after catheter ablation for persistent atrial fibrillation, showing significant advantages compared to various traditional risk scoring systems reported in previous literature. The commonly used CHA_2_DS_2_-VASc score primarily evaluates stroke risk, with its components focusing more on age, comorbidities and thrombosis risk. Although some studies have attempted to use it to predict post-ablation recurrence, its ability to reflect atrial structural and electrophysiological status is limited, resulting in generally poor predictive performance (8, 11). Our results also showed its AUC was only 0.494, almost lacking clinical application value. The APPLE score incorporates factors such as age, persistent atrial fibrillation, renal function, left atrial size and left ventricular ejection fraction during design, theoretically being more aligned with atrial remodeling mechanisms. However, results vary significantly across different research centers (18–20). In our study, its AUC was only 0.506, indicating limited discriminative ability. The MB-LATER score attempted to improve prediction by including variables like early postoperative recurrence, left atrial size and multiple ablation history, but was still limited by sample heterogeneity and difficulties in obtaining some variables (12). In our study, its AUC was 0.663, performing better than the above two scoring systems but overall discriminative ability remained insufficient. In contrast, our study used LASSO regression to screen the most recurrence-related factors from a wide range of candidate variables, and confirmed drug termination, ERAF and SCREA as independent predictors through multivariate regression. These indicators not only cover factors related to atrial electrical remodeling and surgery, but also reflect systemic metabolism and renal function status, enabling the model to more comprehensively capture multidimensional characteristics of recurrence risk. In particular, ERAF has been repeatedly demonstrated as a strong predictor of long-term recurrence after ablation in previous studies, and our study further quantified its risk contribution, with results consistent with previous findings (21–23). On the other hand, drug termination has been rarely reported as a predictor in previous literature. Our study suggests it may reflect the adequacy of ablation operation and atrial electrophysiological response, having protective effects on prognosis, which provides new clues for clinical optimization of perioperative management. SCREA, as a new risk factor, has rarely been involved in previous prediction models. Our study found it closely related to recurrence risk, suggesting that perioperative changes in renal function may affect the atrial electrical remodeling process, expanding previous understanding of recurrence mechanisms.

In this study's prediction model, pharmacologic termination, ERAF, and SCREA were demonstrated to be independent predictors. These three indicators not only showed statistical significance, but also hold important value in pathophysiological mechanisms and clinical management. First, as the strongest risk factor, ERAF had an exceptionally high OR value of 21.188, indicating its extremely significant predictive effect on long-term recurrence. Numerous previous studies have shown that early postoperative recurrence reflects post-ablation electrophysiological remodeling and inflammatory responses, suggesting incomplete elimination of reentry circuits or ectopic foci (24–26). The occurrence of ERAF may be closely related to localized tissue damage from ablation, acute inflammatory responses, and autonomic dysfunction. Its presence often indicates the persistence of atrial electrical remodeling processes, thereby significantly increasing long-term recurrence risk (27–30). Therefore, ERAF is not merely a statistical signal, but rather a clinical manifestation of post-ablation atrial pathophysiological changes, holding significant value for risk assessment and follow-up management. Second, pharmacologic termination demonstrated a protective effect in our study (OR = 0.248), suggesting significantly reduced long-term recurrence risk in patients undergoing pharmacologic rhythm termination. This finding may indicate that pharmacologic termination reflects relatively sufficient ablation procedures while maintaining favorable atrial electrophysiological reversibility. Additionally, the stabilizing effect of medications reduces short-term post-procedural electrical disturbances, thereby improving long-term outcomes (31–33). This differs from conventional perspectives that emphasize the importance of electrical cardioversion or ablation termination, suggesting that pharmacologic termination may also have positive implications. The finding provides new references for clinical selection of termination methods and postoperative medication management. As a novel risk indicator rarely included in previous prediction models, SCREA showed a negative correlation with long-term recurrence risk (OR = 0.976). This parameter reflects dynamic renal function changes, suggesting that ablation procedures and perioperative management may influence systemic metabolic status. Reduced creatinine difference may indicate postoperative renal function improvement with optimized fluid and metabolic environment, thereby diminishing atrial electrical remodeling and trigger factor accumulation (34–36). Conversely, unimproved or increased creatinine difference may suggest underlying systemic inflammation or renal impairment that increases recurrence risk. This discovery first establishes the connection between dynamic renal function changes and AF ablation outcomes, providing new insights into understanding the systemic mechanisms of AF recurrence. Atrial fibrosis and the presence of scar tissue in the left atrium have been increasingly recognized as key factors in the maintenance and recurrence of atrial fibrillation. Our study suggests that the extent of scar tissue is an important predictor of AF recurrence after ablation. Specifically, patients with >30% scar tissue exhibited a significantly higher risk of recurrence, indicating that extensive fibrosis beyond the pulmonary vein isolation area may facilitate re-entrant circuits, contributing to AF persistence. These findings are consistent with previous studies that have shown a strong association between atrial fibrosis and poor long-term outcomes following AF ablation.

The prediction model developed in this study demonstrates significant clinical utility, not only through its superior statistical performance but also by providing an actionable tool for personalized management of persistent atrial fibrillation (AF) patients following catheter ablation. First, the variables selected through LASSO regression all represent routine clinical parameters, including demographic data, laboratory results, procedural characteristics, and early postoperative outcomes. These readily available and cost-effective indicators facilitate model implementation across diverse healthcare settings. Through a nomogram-based visual interface, the model effectively translates complex multivariable regression outcomes into individualized recurrence probability, enabling clinicians to rapidly assess patient prognosis and make more precise decisions regarding follow-up strategies, medication management, and patient education. Second, compared with conventional risk scoring systems, our model demonstrates superior discriminative ability and calibration. ROC analysis revealed significantly higher AUC values than CHA₂DS₂-VASc, APPLE, and MB-LATER scores, indicating more accurate recurrence risk prediction for persistent AF patients post-ablation. Decision curve analysis and clinical impact curves further confirmed the model's ability to provide greater net benefit across various threshold probabilities, suggesting its potential to optimize clinical decision-making by reducing both unnecessary interventions and missed high-risk cases. Moreover, this model enables innovative approaches to personalized post-procedural management. For high-risk patients, clinicians may consider intensified follow-up, early initiation or extended duration of antiarrhythmic therapy, or exploration of novel interventions such as aggressive rhythm control strategies or lifestyle modifications. Conversely, low-risk patients may benefit from reduced follow-up intensity and medication exposure, thereby alleviating healthcare burdens while improving compliance. Importantly, this model establishes a foundation for clinical informatization and intelligent management. Future integration with electronic medical record systems or development as web/mobile applications could facilitate real-time, personalized risk assessment during routine practice, advancing precision medicine in cardiac arrhythmia management.

The interpretation of SCREA as a potential metabolic or renal biomarker for late recurrence is intriguing, but its biological plausibility remains hypothetical. Recent studies have suggested that renal dysfunction, including renal-endothelial dysfunction and inflammation, plays a key role in atrial remodeling and the recurrence of atrial fibrillation (AF) post-ablation. For example, impaired renal function has been linked to an increased recurrence rate after AF ablation (37, 38). Furthermore, endothelial dysfunction and inflammation are important contributors to atrial substrate modification, which may facilitate AF recurrence (39, 40). We hypothesize that a post-procedural increase in creatinine may reflect transient renal or vascular stress, which in turn may exacerbate atrial remodeling and increase the risk of late recurrence. However, further mechanistic studies are required to confirm the role of SCREA as a reliable biomarker for late recurrence in PsAF patients. Additionally, recent advances in artificial intelligence (AI) and machine learning (ML) in the field of electrophysiology have demonstrated their utility in arrhythmia detection, ablation guidance, and prediction of recurrence (41, 42). The integration of AI models with clinical data, including renal function markers like SCREA, holds promise for enhancing the prediction of AF recurrence and optimizing patient management strategies. In summary, while the association between SCREA and late recurrence in PsAF is promising, additional research is needed to explore its mechanistic basis and validate its clinical utility as a biomarker.

While this study has made certain progress, several limitations should be objectively acknowledged and discussed. First, as a single-center retrospective study with a relatively limited sample size, potential selection bias in patient sources and baseline characteristics may affect the model's generalizability and external validity. Second, the follow-up duration was insufficient to fully reflect long-term outcomes. Some patients may experience recurrence beyond the observation period, potentially leading to underestimation of the recurrence rate. Additionally, while the included variables primarily consisted of routine clinical parameters—ensuring good feasibility—they lacked deeper risk factors such as atrial electrophysiological characteristics, imaging markers, or molecular biomarkers. This may somewhat limit the model's predictive accuracy. Moreover, although internal bootstrap resampling and an independent validation set were used for model verification, the external validation sample size remained small and was derived from the same center. Further validation across diverse populations is needed to confirm the model's robustness and generalizability. Finally, the nomogram tool developed in this study remains at the statistical level and has not yet been integrated into clinical information systems or converted into user-friendly computational tools, necessitating further efforts for clinical implementation. In conclusion, the findings should be interpreted cautiously considering these limitations. Future research should refine and optimize the prediction model through multicenter, prospective, large-scale studies incorporating multidimensional variables.

## Data Availability

The original contributions presented in the study are included in the article/Supplementary Material, further inquiries can be directed to the corresponding author.
